# Construction and Evaluation of a Sepsis Risk Prediction Model for Urinary Tract Infection

**DOI:** 10.3389/fmed.2021.671184

**Published:** 2021-05-21

**Authors:** Luming Zhang, Feng Zhang, Fengshuo Xu, Zichen Wang, Yinlong Ren, Didi Han, Jun Lyu, Haiyan Yin

**Affiliations:** ^1^Intensive Care Unit, The First Affiliated Hospital of Jinan University, Guangzhou, China; ^2^Department of Clinical Research, The First Affiliated Hospital of Jinan University, Guangzhou, China; ^3^School of Public Health, Xi'an Jiaotong University Health Science Center, Xi'an, China; ^4^Department of Public Health, University of California, Irvine, Irvine, CA, United States

**Keywords:** urinary tract infection, sepsis, MIMIC III database, prognosis, nomogram

## Abstract

**Background:** Urinary tract infection (UTI) is one of the common causes of sepsis. However, nomograms predicting the sepsis risk in UTI patients have not been comprehensively researched. The goal of this study was to establish and validate a nomogram to predict the probability of sepsis in UTI patients.

**Methods:** Patients diagnosed with UTI were extracted from the Medical Information Mart for Intensive Care III database. These patients were randomly divided into training and validation cohorts. Independent prognostic factors for UTI patients were determined using forward stepwise logistic regression. A nomogram containing these factors was established to predict the sepsis incidence in UTI patients. The validity of our nomogram model was determined using multiple indicators, including the area under the receiver operating characteristic curve (AUC), correction curve, Hosmer-Lemeshow test, integrated discrimination improvement (IDI), net reclassification improvement (NRI), and decision-curve analysis (DCA).

**Results:** This study included 6,551 UTI patients. Stepwise regression analysis revealed that the independent risk factors for sepsis in UTI patients were congestive heart failure, diabetes, liver disease, fluid electrolyte disorders, APSIII, neutrophils, lymphocytes, red blood cell distribution width, urinary protein, urinary blood, and microorganisms. The nomogram was then constructed and validated. The AUC, NRI, IDI and DCA of the nomogram all showed better performance than traditional APSIII score. The calibration curve and Hosmer-Lemeshow test results indicate that the nomogram was well-calibrated. Improved NRI and IDI values indicate that our nomogram scoring system is superior to other commonly used ICU scoring systems. The DCA curve indicates that the DCA map of the nomogram has good clinical application ability.

**Conclusion:** This study identified the independent risk factors of sepsis in UTI patients and used them to construct a prediction model. The present findings may provide clinical reference information for preventing sepsis in UTI patients.

## Background

Sepsis and septic shock are common acute critical diseases with very high mortality rates and which affect millions of people worldwide every year. Early detection and appropriate treatment of sepsis can improve the prognosis (1–3). The early clinical manifestations of sepsis are not specific, the disease progresses, and worsens rapidly, and there is currently no effective treatment. These factors lead to the current high mortality rate of sepsis patients (4, 5). A comprehensive understanding of the pathogenesis of sepsis would allow targeted prevention and treatment to be carried out more effectively. However, there is currently no theoretical explanations of the pathogenesis of sepsis (4). Therefore, in the process of clinical diagnosis and treatment, controlling the risk factors for infection and prevention of sepsis occurrence should be comprehensively studied.

Infection of the lower respiratory tract is the primary cause of sepsis with the highest mortality, but UTI is the fastest-growing cause of sepsis (6). Urosepsis is a serious disease caused by organ failure due to a critical urinary tract infection (UTI) (7), possibly caused by a severe community or hospital-acquired UTI (8). Studies have found that 9–31% of sepsis cases are caused by infections of the urinary and reproductive tracts (9), with urosepsis accounting for 20~30% of all sepsis cases (10). Research have showed that diabetes, C-reactive protein, and calculi are risk factors for urinary sepsis development (11, 12). Nomograms are graphical tools for determining the probabilities of individual experiencing clinical events based on statistical prediction models (13). However, nomograms that predict the sepsis risk in UTI patients have received little attention. The purpose of our study was to develop a nomogram for predicting sepsis risk in UTI patients and thereby guide clinical practice.

## Materials and Methods

### Data Source

All of our data were extracted from the Medical Information Mart for Intensive Care III (MIMIC-III) database, which was established in 2003 under a United States NIH grant by multiple centers. The current (July 2018) version of the MIMIC-III database is version 1.4, which includes data obtained from 2001 to 2012 on more than 58,000 hospitalized patients at the Beth Israel Deaconess Medical Center, including 38,645 adult, and 7,875 neonatal patients. The patient information in this database is anonymous, and so informed consent was not required for this study. The research personnel participated in a series of courses provided by the National Institutes of Health (NIH) and obtained authorization to access the MIMIC-III database after completing the required assessment (certificate number 38601114).

### Study Population

We extracted the required data using structured query language in the Navicat Premium version 11.2.7.0. ICD-9 code 5990 was used to extract patients from the MIMIC-III database who were diagnosed with a UTI. Patients younger than 18 years old or who died within 24 h of entering the ICU were excluded.

The hadm_id identifier of UTI patients was used to extract the following information: age, sex, comorbidities, first laboratory examination results, and APSIII score. Comorbidities (for comorbidities, we evaluated them according to the Elixhauser Comorbidity Index (ECI) in the database, indicate whether the patient was diagnosed with the disease before or after admission.) included congestive heart failure (CHF), hypertension, chronic pulmonary, renal failure, liver disease, metastatic cancer, solid tumors, obesity, urolithiasis, fluid electrolyte disorders, diabetes. The first laboratory test results after admission included levels of white blood cells, neutrophils (NET), lymphocytes (LYM), hemoglobin, hematocrit, platelets, creatinine, urinary white blood cells, urinary red blood cells, urine pH, urinary protein, urinary blood, and urinary ketone, red blood cell distribution width (RDW), and the first pathogenic microorganism culture is positive or negative.

The event outcome was sepsis occurring during hospitalization for UTI. The diagnosis of sepsis is based on the sepsis-3 (4) updated in 2016 by the Society of Critical Care Medicine and the European Society of Intensive Care Medicine, defined as a life-threatening infection combined with an acute increase in Sequential Organ Failure Assessment score (SOFA ≥ 2).

### Statistical Analysis

This study did not include indicators with >20% missing values, and the remaining data were filled using multiple imputation. The “mice” package of R software was used to obtain 10 estimated data sets.

We randomly divided all remaining UTI patients into training (70%) and validation (30%) cohorts. The training cohort was used to construct a nomogram and perform internal validation, and the validation cohort was used to perform external validation. Categorical variables were described as frequency and percentage values, and differences between cohorts were determined using the chi-square or Fisher's exact test. The Shapiro-Wilk test was applied to continuous variables to verify whether they conformed to a normal distribution. Continuous variables were described as mean and standard-deviation values or median and interquartile-range values depending on whether or not they conformed to a normal distribution.

Independent risk factors for sepsis in UTI patients were determined using logistic regression. The variables were screened using forward LN stepwise regression. The determined independent prognostic factors were again analyzed using a logistic regression model, and the results were expressed as odds ratios and 95% confidence intervals (CIs). A nomogram was finally constructed based on the independent prognostic factors to predict sepsis onset in UTI patients.

Multiple indicators were used to internally and externally validate the nomogram. The area under the receiver operating characteristic curve (AUC) was used to evaluate the recognition ability of the nomogram contour map, and this AUC value was compared with that of APSIII. According to the Youden Index, receiver operating characteristic curves were used to determine the optimal cutoff and its sensitivity and specificity. In addition, the integrated discrimination improvement (IDI) and net reclassification improvement (NRI) were used to calculate the performance improvement of the nomogram over the APSIII scoring system. We also constructed a calibration curve and conducted a Hosmer-Lemeshow test to evaluate the calibration of the nomogram. The decision-curve analysis (DCA) curve describes the net benefits and medical interventions from using the nomogram under the guidance of the APSIII, and was used to evaluate the clinical applicability of the nomogram.

R (version 4.0.3) and SPSS (version 24.0) software were used for the statistical analyses, and *P* < 0.05 was considered statistically significant.

## Results

### Baseline Characteristics

The 6,551 patients were divided into 4,585 and 1,965 in the training and validation cohorts, respectively. Females accounted for 61.8 and 60.7% of the UTI patients in the training and validation cohorts, respectively. The median age in both cohorts was 73.0 (60.0, 82.0) years. Patients with diabetes mellitus accounted for 31.6 and 30.1% of those in the training and validation cohorts, respectively, while patients with urinary stones accounted for 0.5 and 0.4% and those with hypertension accounted for 59.1 and 58.9%. The median APSIII score in both cohorts was 45.0 (34.0, 59.0). From the laboratory test results, patients with proteinuria accounted for 67.5 and 69.3% of those in the training and validation cohorts, respectively, hematuria patients accounted for 73.2 and 73.8%, and sepsis patients accounted for 15.1 and 16.1%. The remaining baseline characteristics are listed in Table 1.

**Table 1:** Patient characteristics.

**Variable**	**Validation cohort**	**Training cohort**	***p***
*N*	1,965	4,585	
Sex (%)			
Male	772 (39.3)	1,752 (38.2)	0.428
Female	1,193 (60.7)	2,833 (61.8)	
Age	73.00 (60.00, 82.00)	73.00 (60.00, 82.00)	0.378
APSIII	45.00 (34.00, 59.00)	45.00 (34.00, 59.00)	0.573
Comorbidities			
Congestive heart failure (%)			
No	1,212 (61.7)	2,909 (63.4)	0.184
Yes	753 (38.3)	1,676 (36.6)	
Hypertension (%)			
No	808 (41.1)	1,874 (40.9)	0.874
Yes	1,157 (58.9)	2,711 (59.1)	
Chronic pulmonary (%)			
No	1,484 (75.5)	3,466 (75.6)	0.975
Yes	481 (24.5)	1,119 (24.4)	
Diabetes (%)			
No	1,374 (69.9)	3,138 (68.4)	0.246
Yes	591 (30.1)	1,447 (31.6)	
Renal failure (%)			
No	1,544 (78.6)	3,629 (79.1)	0.624
Yes	421 (21.4)	956 (20.9)	
Liver disease (%)			
No	1,738 (88.4)	4,112 (89.7)	0.150
Yes	227 (11.6)	473 (10.3)	
Metastatic cancer (%)			
No	1,870 (95.2)	4,346 (94.8)	0.565
Yes	95 (4.8)	239 (5.2)	
Solid tumors (%)			
No	1,917 (97.6)	4,456 (97.2)	0.444
Yes	48 (2.4)	129 (2.8)	
Obesity (%)			
No	1,847 (94.0)	4,315 (94.1)	0.900
Yes	118 (6.0)	270 (5.9)	
Urolithiasis (%)			
No	1,957 (99.6)	4,561 (99.5)	0.671
Yes	8 (0.4)	24 (0.5)	
Fluid electrolyte disorders (%)			
No	1,095 (55.7)	2,628 (57.3)	0.244
Yes	870 (44.3)	1,957 (42.7)	
First laboratory test			
WBC (K/uL)	10.50 (7.70, 14.80)	10.50 (7.50, 14.80)	0.241
Lymphocytes (%)	11.00 (6.00, 17.40)	11.00 (6.30, 17.60)	0.387
Neutrophils (%)	80.90 (72.50, 87.40)	81.30 (72.80, 87.80)	0.299
Hematocrit (g/dL)	33.90 (29.70, 38.30)	34.10 (30.00, 38.50)	0.124
Hemoglobin (g/dL)	11.30 (9.80, 12.80)	11.40 (9.90, 12.90)	0.125
RDW (%)	14.80 (13.80, 16.50)	14.90 (13.80, 16.50)	0.919
Platelet (K/uL)	235.00 (173.00, 311.00)	240.00 (175.00, 319.00)	0.095
Creatinine (mg/dL)	1.10 (0.80, 1.80)	1.10 (0.80, 1.80)	0.656
Urine RBC (#/hpf)	5.00 (2.00, 20.00)	5.00 (2.00, 20.00)	0.714
Urine WBC (#/hpf)	10.00 (3.00, 50.00)	10.00 (2.00, 50.00)	0.248
Urine Ph	5.50 (5.00, 6.50)	5.50 (5.00, 6.50)	0.299
Urine blood (%)			
Negative	514 (26.2)	1,227 (26.8)	0.634
Positive	1,451 (73.8)	3,358 (73.2)	
Urine protein (%)			
Negative	604 (30.7)	1,491 (32.5)	0.165
Positive	1,361 (69.3)	3,094 (67.5)	
Urine ketone (%)			
Negative	1,259 (64.1)	3,020 (65.9)	0.170
Positive	706 (35.9)	1,565 (34.1)	
Microorganism (%)			
Negative	1,396 (71.0)	3,175 (69.2)	0.155
Positive	569 (29.0)	1,410 (30.8)	
Outcome			
Sepsis (%)			
No	1,633 (83.1)	3,892 (84.9)	0.075
Yes	332 (16.9)	693 (15.1)	

### Nomogram Construction

**Figure 1:**
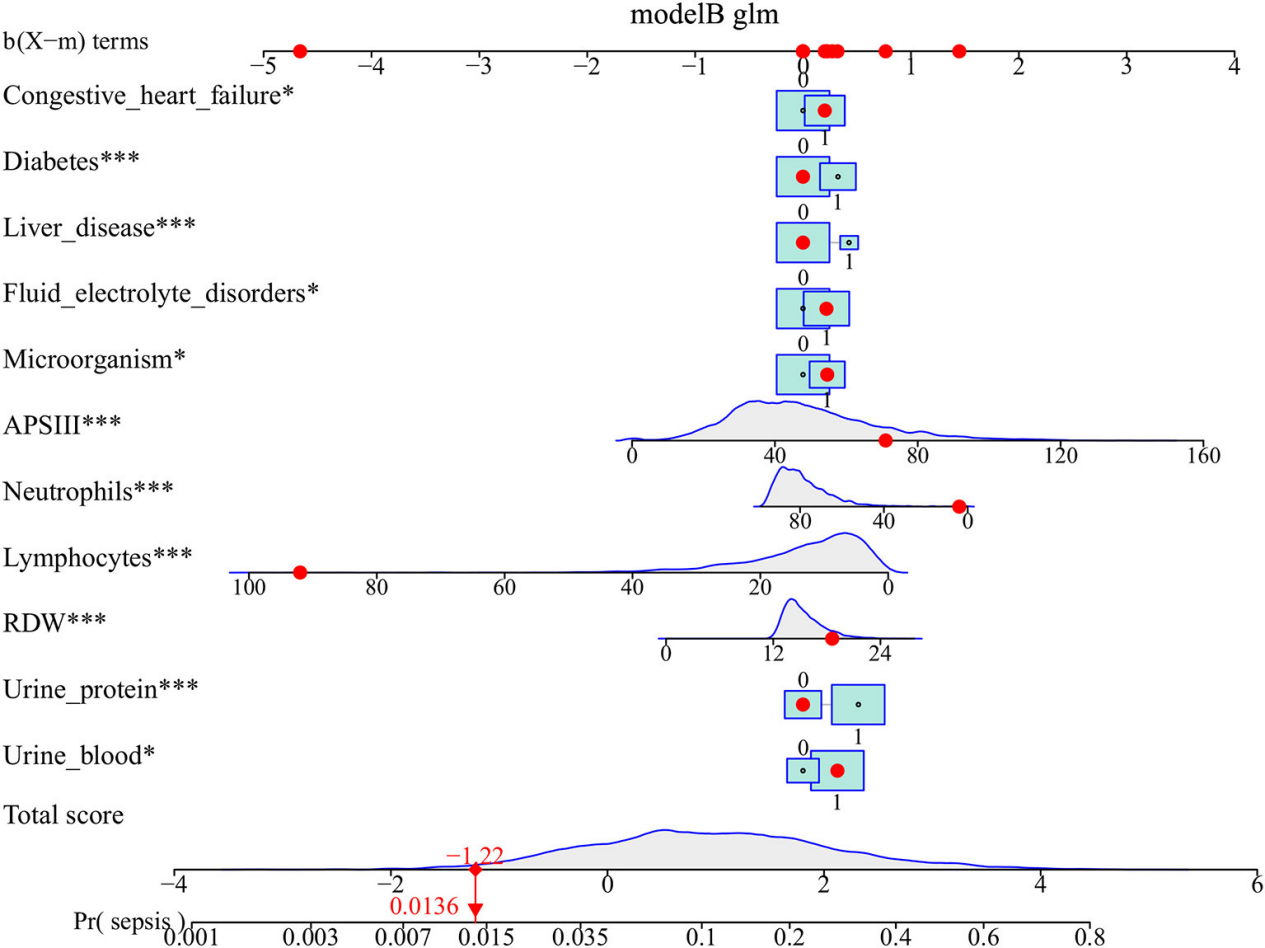
Nomogram predicts the probability of sepsis in patients with urinary tract infection. When using the nomogram, drawing a vertical line from each variable upward to the terms and then recording the corresponding points. The point of each variable was then summed up to obtain a total score that corresponds to a predicted probability of sepsis at the bottom of the nomogram. The red dots represent the indicators of a patient in our study population and the corresponding probability of sepsis. For comorbidities, 0 means that the patient does not have this comorbiditiy, 1 means that the patient has. For laboratory test results, 0 means negative, and 1 means positive. *p < 0.05, and ***p < 0.001 in the multivariate logistic analysis.

The above-mentioned variables were screened using forward LN stepwise regression. CHF, diabetes, liver disease, fluid electrolyte disorders, APSIII, NET, LYM, RDW, urinary protein, urinary blood, and microorganisms were established as independent risk factors for sepsis in UTI patients during hospitalization. The sepsis risk was 1.382-fold higher in diabetic than non-diabetic patients (95% CI = 1.151–1.656), 1.534-fold higher in patients with than without liver disease (95% CI = 1.186–1.973), 1.668-fold higher in patients with than without proteinuria (95% CI = 1.323–2.116), and 1.374-fold higher in patients with than without hematuria (95% CI = 1.081–1.759). The protective factors for sepsis were NET and LYM (see Table 2). These results were used to construct a nomogram for estimating the sepsis risk in UTI patients during hospitalization (Figure 1).

**Table 2:** Independent risk factors for sepsis in UTI patients in the multivariate logistic analysis.

	**OR**	**95% CI**		***p***
Congestive heart failure				0.030
No	Reference			
Yes	1.22	1.02	1.47	
Diabetes				<0.001
No	Reference			
Yes	1.38	1.15	1.66	
Liver disease				
No				
Yes	1.53	1.19	1.97	<0.001
Fluid electrolyte disorders				
No				
Yes	1.24	1.04	1.49	0.019
Microorganism				
Negative	Reference			
Positive	1.25	1.04	1.51	0.018
APSIII	1.03	1.03	1.04	<0.001
Neutrophils (%)	0.98	0.97	0.99	<0.001
Lymphocytes (%)	0.94	0.93	0.96	<0.001
RDW (%)	1.09	1.04	1.13	<0.001
Urine blood				
Negative	Reference			
Positive	1.37	1.08	1.76	0.010
Urine protein				
Negative	Reference			
Positive	1.67	1.32	2.12	<0.001

### Nomogram Validation

**Figure 2:**
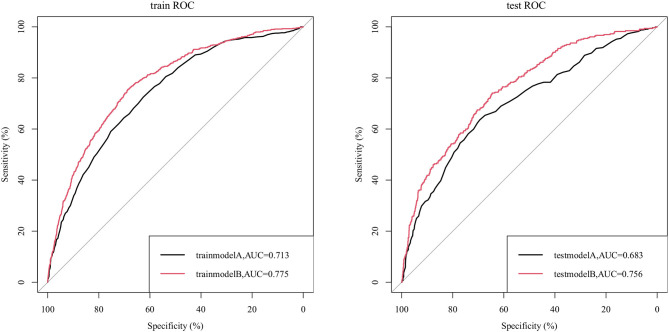
ROC curves for the APSIII model (model A) and the nomogram (model B).

We compared the ability to predict sepsis in UTI patients between the nomogram and the APSIII scoring system. Figure 2 indicates that the AUC values of the nomogram were 0.775 (95% CI = 0.757–0.792) and 0.756 (95% CI = 0.730–0.784) for the training and validation cohorts, respectively, which were higher than those of the APSIII scoring system. The optimal cutoff for the nomogram in the training cohort was 0.144, and the sensitivity and specificity were 0.706 and 0.701, respectively. In the validation cohort, the optimal cutoff was 0.132, the sensitivity was 0.730, and the specificity was 0.784. Compared with the APSIII system, the NRI values of the nomogram were 0.306 (95% CI = 0.242–0.419) and 0.306 (95% CI = 0.236–0.406) in the training and validation cohorts, respectively; the corresponding IDI values were 0.021 (95% CI = 0.012–0.030) and 0.034 (95% CI = 0.017–0.051). These findings indicate that our nomogram has better recognition ability and is superior to other commonly used scoring systems.

**Figure 3:**
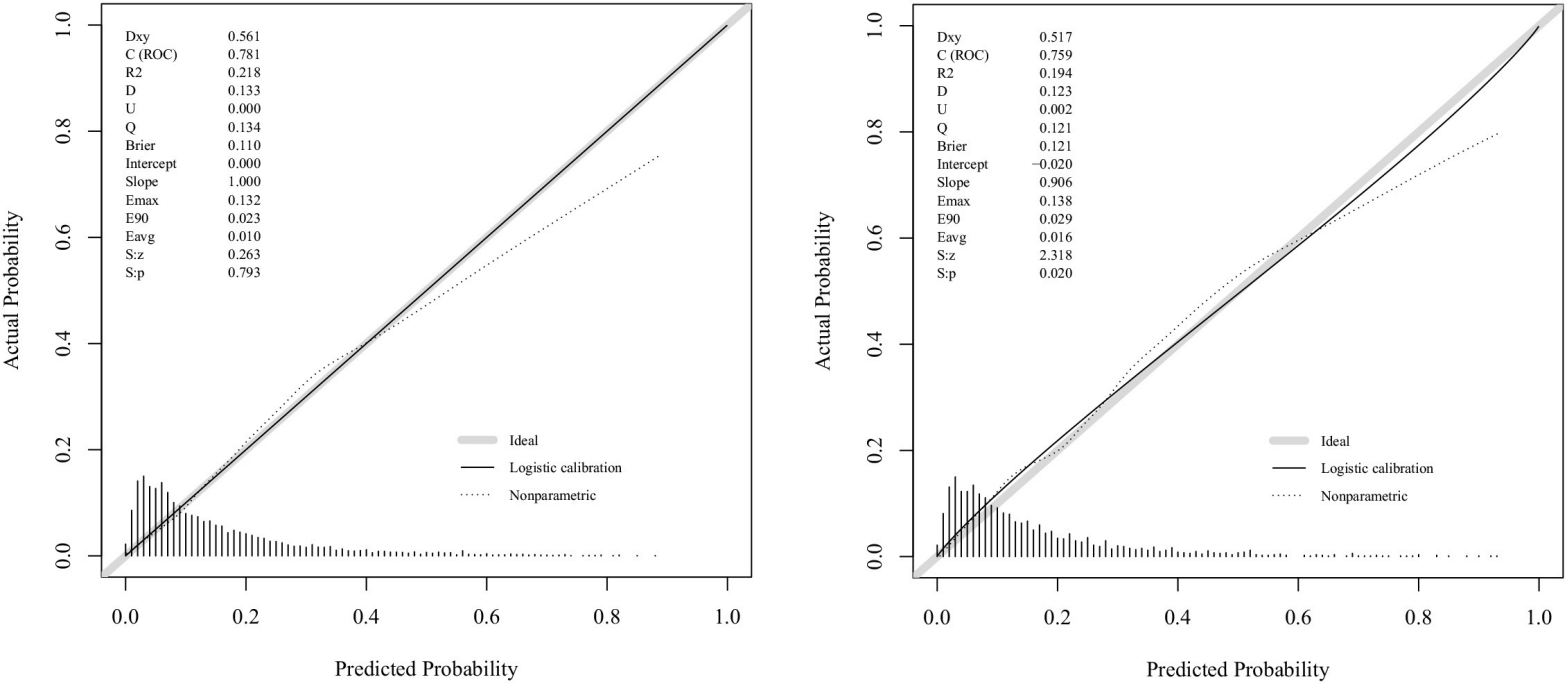
Calibration curves for the validation cohort and the training cohort.

**Figure 4:**
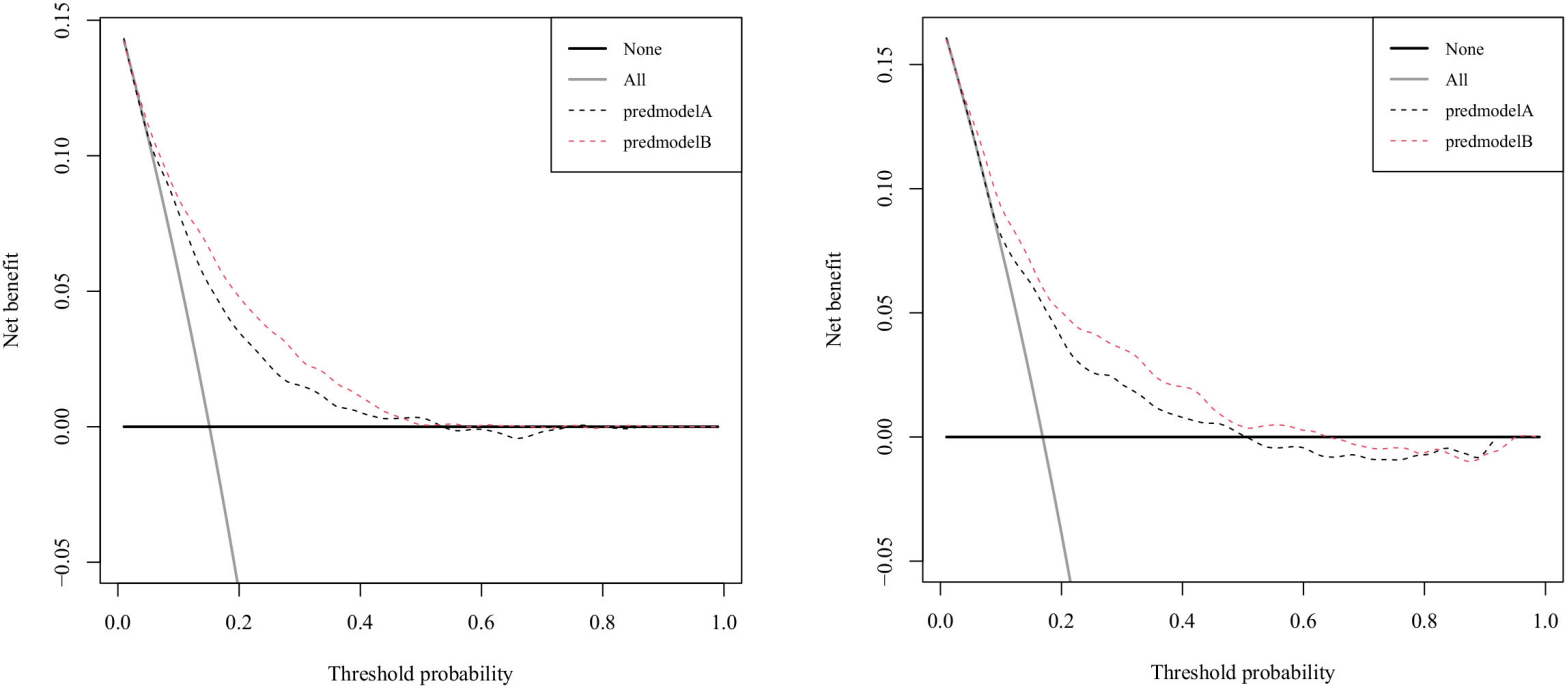
Decision-curve analysis of the validation cohort and the training cohort. Model A represents the APSIII model, and model B represents the nomogram.

Figure 3 displays the correction curves of the nomogram. The correction curves of the training and validation cohorts were all almost diagonal, and the Hosmer-Lemeshow test results indicated the absence of statistical significance (training cohort: χ^2^ = 6.950, *P* = 0.642; validation cohort: χ^2^ = 11.823, *P* = 0.223), indicating that the nomogram provided a good fit to the data. Finally, we drew a DCA curve to illustrate the clinical applicability of the nomogram and compared it with the APSIII system (Figure 4). Clinical interventions guided by our nomogram had a higher net benefit than other scoring systems when the threshold probability was between 0.1 and 0.8 in both cohorts.

## Discussion

Our study indicated that CHF, diabetes, liver disease, fluid electrolyte disorders, APSIII, NET, LYM, RDW, urinary protein, urinary blood, and microorganisms are independent risk factors for sepsis in UTI patients. These results were used to construct a nomogram for estimating the sepsis risk in UTI patients during hospitalization. The validity of our nomogram model was determined using multiple indicators, including AUC, correction curve, Hosmer-Lemeshow test, IDI, NRI, and DCA. We determined the best cutoff value according to the Yoden index, and also considered the sensitivity and specificity. In practical application, the choice of cutoff value could be weighed according to the risk of misdiagnosis and missed diagnosis.

In some populations, such as critically ill patients, the fatality rate for urine-derived sepsis is 25–60% (14). The occurrence of sepsis can be reduced through early risk assessment, reasonable antibiotic treatment, and timely targeted treatments (15). Among the complications, CHF, diabetes, liver disease, and electrolyte disturbances were all associated with sepsis. Diabetes was found to be the most common complication relating to UTI (16). The mechanism involves diabetic patients abnormally metabolizing sugar, fat, and protein, and having reduced protein synthesis and accelerated overall metabolism, resulting in reduced synthesis of immunoglobulin, antibodies and complement, reduced LYM conversion rate, and impaired humoral immune function (17). Leukocyte migration, chemotaxis, phagocytosis, and bactericidal ability in the blood were also significantly reduced, and cell-mediated immune function was also reduced. Urinary sugar is a good culture medium, provides favorable conditions for bacterial growth and reproduction, and is also conducive to fungal growth (18). The mechanism of agricultural sepsis is therefore greatly enhanced in diabetic patients.

Our study showed an increased risk of sepsis in patients with the first positive microbial culture. The pathogen enters the urinary system through retrograde, hematogenous, or lymphatic pathways. Urine-derived sepsis is caused by the pathogen developing further after it enters the blood *via* the urethra. Patients with pathogen cultures present for the first time should receive clinical attention. Appropriate specimens should be retained for etiological culture before antibiotic use. Antimicrobial agents should include all possible pathogenic bacteria in the initial stage of anti-infective therapy.

NET is one of the important components for the host to fight infection (19), and various mechanisms can be used to enhance the protective immune response (20). LYM is an important component in the immune response of the body, and are an important defense mechanism in biological systems (21). Our results indicated that both NET and LYM were protective factors and that increases in these substances decrease the probability of urine-derived sepsis.

RDW is a clinically-accessible parameter that reflects variations in the size of red blood cells and has mostly being used in the diagnosis of circulatory diseases (22). RDW is currently considered to be a strong independent risk factor for human mortality, and many studies have investigated the cardiovascular, cerebrovascular, renal, and other aspects of this parameter (23–26). Some studies suggest that RDW has important prognostic value, and can predict hospital and 4-year mortality in critically ill patients (27). RDW is strongly weighted as a risk factor for sepsis in UTI patients in the present nomogram. One study indicated that (28) there is a strong, positive, and independent association between RDW and traditional biomarkers of inflammation, possibly because inflammation reduces the survival rate of red blood cells, leading to differences in red blood cell volumes, and an increase in size heterogeneity among red blood cells. Other studies have indicated that oxidative stress may lead to increases in RDW by increasing erythrocyte turnover, therefore resulting in an association between cell-size inequality and human pathology (29). Patients with increased RDW values should therefore receive specific attention in order to improve their clinical outcomes.

Hematuria is divided into gross and microscopic hematuria, which are common symptoms among UTI patients (30). Proteinuria is positive in 63–83% of culture-confirmed UTI cases (31). Our results suggest that hematuria and proteinuria are risk factors for sepsis. However, due to the qualitative nature of this study, no conclusions on the quantitative issues of proteinuria and hematuria can be drawn, and the presence of early renal lesions promoting sepsis occurrence remains unclear.

Clinical prediction models were used to investigate the relationship between future outcome events and baseline health status in patients with specific conditions. These can integrate the results of traditional analyses, simplify them through more visually based and convincing presentations, and predict the occurrence probability of certain outcome events through scoring systems. Sepsis and septic shock are major health care problems that affect millions of people around the world every year, and building predictive models for sepsis is clinically important. Some scholars have constructed a nomogram to predict the probability of sepsis after percutaneous nephrolithotomy, and the results showed that patients with complex stones and positive bacteriuria had a significantly higher risk of sepsis after surgery (32). This is a study on urolithiasis, and our study included all patients with UTI in the database, using the history of urolithiasis as one of the study variables. Moreover, we use new indicator such as RDW for the first time to predict the probability of sepsis in patients with urinary tract infection. Other study developed a predictive model that could provide an early risk assessment of sepsis in patients undergoing major hepatobiliary and pancreatic surgery (33). In addition, there are many prognostic models for sepsis patients, for example, one study constructed a predictive model of the 30-day risk of death in patients with sepsis-associated encephalopathy that could be used to assess their prognosis (34). Unlike these studies, to the best of our knowledge, we have constructed the first model that can predict the probability of sepsis in patients with UTI, based on the outcome of the patient's first laboratory examination and comorbidities. This provides a basis for the clinical treatment of patients with UTI. Doctors can use the scoring results of the model to communicate with them and help patients understand the severity of the disease, so that they can jointly make treatment plans and improve their cooperation to prevent the occurrence of sepsis to the greatest extent.

## Strengths and Limitations of the Study

The strength of this study is that we used the MIMIC-III database, a public database containing a large amount of critically ill patient information, which provides strong evidence for our results. Moreover, we developed a nomogram to assess the probability of sepsis by laboratory tests and complications after admission to the ICU in patients with UTI, and demonstrated that the model was effective, something that had not been done before. This study also has some limitations. Although, the number of patients is large, it is a single-center study and lacks external validation.

## Conclusion

This study identified the independent risk factors of sepsis in UTI patients and used them to construct a prediction model based. The present findings may provide clinical reference information for preventing sepsis in UTI patients.

## Data Availability Statement

The datasets presented in this study can be found in online repositories. The names of the repository/repositories and accession number(s) can be found below: The data were available on the MIMIC-III website at https://mimic.physionet.org/, http://dx.doi.org/10.13026/C2XW26.

## Ethics Statement

The study was an analysis of a third-party anonymized publicly available database with pre-existing institutional review board (IRB) approval. Data extracted from the MIMIC III database do not require individual informed consent because MIMIC III database research data is publicly available and all patient data are de-identified.

## Author Contributions

LZ created the study protocol, performed the statistical analyses, and wrote the first manuscript draft. FZ conceived the study and critically revised the manuscript. FX assisted with the study design and performed data collection. ZW assisted with study coordination and helped draft the manuscript. YR assisted with data collection and manuscript editing. DH confirmed the data and assisted with the statistical analyses. JL assisted with manuscript revision and data confirmation. HY contributed to data interpretation and manuscript revision. All authors read and approved the final manuscript.

## Conflict of Interest

The authors declare that the research was conducted in the absence of any commercial or financial relationships that could be construed as a potential conflict of interest.
